# Reducing corruption in a Mexican medical school: impact assessment across two cross-sectional surveys

**DOI:** 10.1186/1472-6963-11-S2-S13

**Published:** 2011-12-21

**Authors:** Sergio Paredes-Solís, Ascensio Villegas-Arrizón, Robert J Ledogar, Verónica Delabra-Jardón, José Álvarez-Chávez, José Legorreta-Soberanis, Elizabeth Nava-Aguilera, Anne Cockcroft, Neil Andersson

**Affiliations:** 1 Centro de Investigación de Enfermedades Tropicales (CIET), Universidad Autónoma de Guerrero, México; 2 CIETinternational, 511 Avenue of the Americas, #132, New York, NY 10011, USA; 3 Facultad de Medicina, Universidad Autónoma de Guerrero, México; 4 CIET Trust Botswana, PO Box 1240, Gaborone, Botswana

## Abstract

**Background:**

Corruption pervades educational and other institutions worldwide and medical schools are not exempt. Empirical evidence about levels and types of corruption in medical schools is sparse. We conducted surveys in 2000 and 2007 in the medical school of the Autonomous University of Guerrero in Mexico to document student perceptions and experience of corruption and to support the medical school to take actions to tackle corruption.

**Methods:**

In both 2000 and 2007 medical students completed a self-administered questionnaire in the classroom without the teacher present. The questionnaire asked about unofficial payments for admission to medical school, for passing an examination and for administrative procedures. We examined factors related to the experience of corruption in multivariate analysis. Focus groups of students discussed the quantitative findings.

**Results:**

In 2000, 6% of 725 responding students had paid unofficially to obtain entry into the medical school; this proportion fell to 1.6% of the 436 respondents in 2007. In 2000, 15% of students reported having paid a bribe to pass an examination, not significantly different from the 18% who reported this in 2007. In 2007, students were significantly more likely to have bribed a teacher to pass an examination if they were in the fourth year, if they had been subjected to sexual harassment or political pressure, and if they had been in the university for five years or more. Students resented the need to make unofficial payments and suggested tackling the problem by disciplining corrupt teachers. The university administration made several changes to the system of admissions and examinations in the medical school, based on the findings of the 2000 survey.

**Conclusion:**

The fall in the rate of bribery to enter the medical school was probably the result of the new admissions system instituted after the first survey. Further actions will be necessary to tackle the continuing presence of bribery to pass examinations and for administrative procedures. The social audit helped to draw attention to corruption and to stimulate actions to tackle it.

## Background

Corruption is a worldwide phenomenon pervading many types of institutions, including those in the education sector. Education is conceivably the most important sector for launching efforts to prevent corruption [1]. In higher education, increased competition among students has led to new opportunities for corrupt practices [2]. Rumyantseva classified educational corruption by area (academic or services), and by the people involved in the exchange (student-faculty, student-administrator, and student-staff) [3]. These classifications refer to bribery in the course of delivering educational services and do not include other types of corruption such as political and administrative corruption or academic fraud [4].

Corruption in the education sector is not limited to strictly academic institutions. Offers to sell grades, fake degrees, and false accreditation and certification licenses are increasingly promoted by individuals or companies in several countries [5]. A review by Heyneman on corruption in higher education in Bulgaria, Moldova, Croatia and Serbia, found various forms of corruption across different disciplines. Faculties of economics, law and medical sciences were the most corrupt [6]. Bribery at lower education levels may be predictive of corrupt behaviour at higher levels. A survey in Ukraine reported that those who gave bribes for their final exams in secondary education were more likely to give them for admission to the next education level [7].

The little research done on ethical issues that might have a negative impact on the professionalism of future physicians has tended to focus on medical student behaviour in general [8], examining corrupt practices only in this context [9]. Misconduct of medical students covers a whole range of behaviour from signing the attendance list for an absent classmate, through to more seriously corrupt practices like paying a bribe for passing an examination.

The strong social position of the university professor makes it easy for him or her to influence student behaviour inappropriately in a variety of ways such as persuading them to vote for a particular university faction or to submit to sexual harassment.

A Mexican newspaper recently reported the discovery by health authorities that the text of the national examination for a medical residency was being sold [10]. A subsequently published comment referred to this as a long-standing practice [11]. During residency training for medical specialities, the young physician is offered gifts by pharmaceutical companies [12]. This has generated debates about the degree of influence that these ‘gifts’ have on the physician’s impartiality when prescribing medicines [13]. There have been calls for the establishment of norms to regulate these gifts, and for a standard of unbiased pharmaceutical information in the curricula of medical schools [14]. Some programmes to diminish the effects of the pharmaceutical representatives on medical students are currently being developed [15].

It is plausible that doctors who behaved dishonestly as medical students will continue such behaviour with their patients and in their jobs [16]. Unofficial payments among government health workers have been found to occur mainly among medical doctors [17,18]. The linkage between exposure to unofficial payments at the medical schools and the occurrence of unofficial payments among health professionals still needs further research.

Recourse to the influence of private contacts and paying for admission into medical school are a concern in at least certain countries [19,20]. High demand for entry into medical school opens a window for corruption. In Slovakia, 45% of households with university students responded that it is definitely not possible to be accepted in medical school without giving a bribe [21]. Hrabak in 2004 reported that 0.7% of the medical students in Zagreb paid for passing an examination [9]. In Serbia 5% of medical students paid a teacher for passing an exam and 27% bribed a faculty member for a grade [3]. According to the authors of a survey of 203 physicians in Poland in 2001, there was a generalised belief that medical school teachers were among the groups that benefited most from corruption [22].

We conducted two cross-sectional surveys in the Autonomous University of Guerrero, the first in 1999-2000 and the second in 2007. The main objective of the surveys was to measure students' perception and experience of the university’s integrity and to propose actions to improve it.

## Methods

The research was approved by the CIET Mexico ethics committee. We obtained informed consent from all participants, who understood they were free to refuse to participate in the study and to refuse to answer any questions they chose not to.

In both 2000 and 2007, the students who were present in the classes during the day of the survey completed a self-administered questionnaire in their classrooms, in the presence of the researchers but without the presence of the teacher. Both surveys covered 41 preparatory schools and 30 university colleges, including the medical school. The questions in the two surveys were the same and enquired about: age and sex; years of study in the medical school; perception of the main academic and administrative problems in the medical school; unofficial payments for registration (entry), to pass an examination, or for administrative services; and suggestions about how to deal with corruption.

### Analysis

Data operators entered data twice with validation, using Epi Info software. Analysis relied on CIETmap open source software [23] which incorporates an interface to the widely used public domain R software. We examined the change in unofficial payments between 2000 and 2007 and factors related to making unofficial payments in 2007. Bivariate and then multivariate analysis was based on the Mantel-Haenzsel procedure [24]. Multivariate analysis began with saturated models including all variables potentially associated with the outcome, and stepped down to final models including only variables that remained significantly associated with the outcome. The significance of associations is described by the adjusted Odds Ratio (ORa) and the 95% confidence interval (CI) around the ORa.

To compare reported payments in 2000 with reported payments in 2007, we converted the figures into equivalent days of work according to the average official minimum wage for the geographical zone of Acapulco city: 33.33M$ in 2000 and 46.09 M$ in 2007 [25].

After each survey, focus groups of students discussed the key quantitative results, mechanisms of bribes transfer, priority problems in the medical school, what should be done to reduce corruption in the school, and who should lead the effort to achieve solutions.

We distributed the results from the 2000 survey to students and university workers, and presented the final report of the survey to the University Council, the highest governing body of the university, framed in terms of the need for institutional reforms to improve academic quality at the university.

## Results

We interviewed 46% of the medical school’s student body (725/1566) in 2000 and 44% in 2007 (436/986). The lower number of students on the 2007 survey reflects the enrolment policy, established in 2002, which decreased the number of students admitted to the medical school. Of the 1095 students taking classes in the academic session (morning) of the survey in 2000, 164 were in a class outside the medical school, and 206 (19%) were “habitual absentees”; all the 725 actually present completed the questionnaire. Of 615 students taking classes in the morning session of the 2007 survey, 120 were in a class outside the medical school, and 59 (10%) were “habitual absentees”; all the 436 actually present completed the questionnaire. Some 56% (398/706) of the respondents who indicated their sex were male in 2000 and 60% (260/430) in 2007.

Some 8% (59/717) of medical students responding to the 2000 survey reported corruption as the medical school's main academic problem (Table 1). That proportion increased to 22% (94/418) in the 2007 survey: a medical student in the 2007 survey was three times more likely to affirm that corruption was the main problem of the faculty compared with a medical student in the 2000 survey (OR 3.14, 95% CI 2.24–4.41). Absenteeism among teachers, another expression of corruption, was rated as the main academic problem by 12% of students in 2000 and 9% in 2007. Corruption in the school’s administrative processes was perceived almost equally in the two surveys; 13% in 2000 and 12% in 2007 considered it the main administrative problem. The most common “main academic problem” cited in 2000 was excessive political discussions (58% of students), while in 2007 it was low student performance (32% of students).

**Table 1:** Medical students' perception of the main problem in their school

	2000 survey	2007 survey
*Main academic problem*	*n*=*717*	*n*=*418*
Excessive political discussions	413 (58%) †	110 (26%) †
Absenteeism of teachers	83 (12%)	39 (9%)
Low academic level	87 (12%)	40 (10%)
Low student performance	75 (11%) †	135 (32%) †
Corruption	59 (8%) †	94 (22%) †
*Main administrative problem*	*n*= *710*	*n*= *429*
Lack of equipment	399 (56%) †	168 (39%) †
Overcrowding of students	109 (15%) †	18 (4%) †
Corruption	90 (13%)	50 (12%)
Student services management	62 (9%) †	139 (32%) †
Cleanliness of the school	50 (7%)	54 (13%)

† = Difference between 2000 and 2007 significant at the 5% level

Table 2 shows the numbers and proportions of students who reported making unofficial payments in the two surveys. The proportion of students that reported having paid unofficially for registration in the medical school dropped from 6% (44/712) in 2000 to 1.6% (7/436) in 2007. A student in the medical school in 2000 was four times as likely to report having paid unofficially for their registration into the school compared with a student in the medical school in 2007 (OR 4.02, 95% CI 1.9 – 8.5).

**Table 2:** Students who reported making unofficial payments in the medical school, by year of the four-year course

	Number (%) who made an unofficial payment				
*2000 survey*	*First year* (*n*= *367*)	*Second year* (*n*=*152*)	*Third year* (*n*=*98*)	*Fourth year* (*n*=*95*)	*All years* (*n*=*712*)
For admission to the medical school	17 (5)	6 (4)	9 (9)	12 (12)	44 (6)
To pass an examination	7 (2)	32 (21)	27 (28)	37 (40) †	104 (15)
For an administrative procedure	27 (8)	9 (6)	13 (14)	14 (15)	63 (9)
*2007 survey*	*First year* (*n*= *145*)	*Second year* (*n*= *100*)	*Third year* (*n*=*103*)	*Fourth year* (*n*=*88*)	*All years* (*n*= *436*)
For admission to the medical school	1 (1)	0	4 (4)	2 (2)	7 (1.6)
To pass an examination	4 (3)	1 (1)	24 (24)	47 (57) ‡	76 (18)
For an administrative procedure	3 (2)	8 (8)	8 (8)	14 (16)	33 (8)

† = *X*^2^ Test of trend 111.7, df 3, p<0.0001

‡ = *X*^2^ Test of trend 122.0, df 3, p<0.0001

Amongst the students in all four years of the course together, the proportion who reported paying to pass an examination did not differ significantly between 2000 (15%) and 2007 (18%). In both 2000 and 2007, the proportion of students who reported they had ever bribed a teacher to pass an examination was higher in the later years of the course, and was highest in the fourth (final) year (Table 2) . The trend was significant in both 2000 and 2007 (*X*^2^ test of trend 111.7, 3 df, p<0.0001 in 2000, and 122.0, 3 df, p<0.0001 in 2007). The proportion who reported paying for an administrative procedure (such as enrolment certificate or student ID) was similar in 2000 (9%) and 2007 (8%).

In the 2007 survey, four variables were independently associated with ever having paid a bribe to a teacher to pass an examination (Table 3). The strongest association was with being a fourth year student. Students who reported having been subjected to sexual harassment or political pressure were also more likely to have paid a teacher to pass an examination. Those students who had been at the university five years or more were more likely to have paid a bribe to pass an examination.

**Table 3:** Final model of variables associated with unofficial payments from medical students to teachers for passing an examination, 2007 survey

Variable	Crude Odds Ratio	Adjusted Odds Ratio	95% confidence interval
Student in fourth year	14.9	9.6	5.4 – 16.9
Sexual harassment by teachers	3.6	3.2	1.4 – 7.4
Political harassment by teachers	3.6	3.2	1.6 – 6.3
Enrolled in university >5 yrs	4.7	2.6	1.4 – 4.9

The following variables were included in the initial model:

Sex of the student

Age of the student

Course year of the student

Years of enrolled in the university

Perception of corruption as main problem in the university

Perception of corruption as main academic problem in the medical school

Sexual harassment by teachers

Political harassment by teachers

Unofficial payments for an administrative procedure

Unofficial payment for admission to the medical school

As shown in Table 4, the average amount paid by students who had paid unofficially for registration at the medical school (in terms of days at the minimum wage) was higher in the 2000 survey than in the 2007 survey, but the difference was not significant at the 5% level (t test 1.49, 34 df, p =0.14). On the other hand, the reported bribe to a teacher for passing an examination increased significantly between the 2000 survey and the 2007 survey (t test 3.42, 148 df, p =0.0008). The average amount paid for bribes for administrative procedures did not change significantly between the two surveys (t test 1.09, 67df, p =0.28).

**Table 4:** Average amount paid unofficially (converted to days of work at the minimum wage)

Item	2000 survey			2007 survey			
	n=	mean	SD	n=	mean	SD	p value †
For admission to the medical school	30	149	137	6	64	34.5	0.14
For passing an examination	83	8.3	9.4	67	14.6	13.1	0.0008
For an administrative request at the school	48	22.2	76.6	21	3.9	4.7	0.28

SD = standard deviation

† = p value from *t* test

### Suggested actions to stop bribes

The questionnaire asked for suggestions for how corruption in the medical school could be reduced. In 2000, some 53% of students (377/712) said those teachers who ask for a bribe should be dismissed from their employment. Administrative sanctions (such as salary penalties) were suggested by 24% (174/702) of students; 14% (101/712) wanted public disclosure of the teacher’s name; and 8% (60/712) said nothing could be done. In the 2007 survey, 44% of students (185/424) believed dismissing the teacher involved was the way to stop bribes. The other suggestions in 2007 were: administrative sanctions 31% (130); disclosure 19% (79) and 7% (30) said nothing could be done.

Student focus groups in 2000 considered the frequency and types of corruption in the medical school revealed by the survey were realistic. They noted that political harassment and poor preparation of classes by teachers were also a form of corrupt behaviour. They felt that poor preparation of classes by teachers was the most important problem and suggested this should be prioritised by the university authorities. They felt excessive demand for admission to the school was the main reason students paid for their registration. They said the most common administrative procedures for which students had to make unofficial payments were: obtaining a student identification card and certificate of enrolment, registration for a re-examination, and fast access to ones grades. They said some teachers were notorious for demanding bribes to pass examinations, even establishing ‘tariffs’ for different examinations. Some teachers linked political harassment and selling of grades. Bad behaviour by teachers, who usually have two or three jobs in addition to teaching in the medical school, was fostered by lack of administrative controls and sanctions. The students were optimistic that the medical school could improve the system and reduce corruption and they considered students could support this action if they simply stopped bribing.

### Action by the medical school

After the results of the 2000 survey were presented to the University Council, the Faculty of Medicine introduced three relevant policy reforms in 2002: reduction in the number of students, an independent evaluation for selection of new students, and collective examination of students by department heads rather than by individual teachers.

## Discussion

### Limitations

We conducted our survey during an ordinary class day at the medical school and administered the questionnaire to all students who were present during that morning session. The number of students who participated in the survey was less than half the total student body in each survey. However, most of those not included were not present in the school at the time of the survey because they did not have classes in the school, so this is not likely to have introduced a bias. There was a lower proportion of “habitually absent” students in 2007 compared with 2000. These habitual absentees may well be students who expected to be able to complete their course by paying a bribe to pass examinations, so their absence in the sample may have led to an underestimate of the level of bribes, with this being more apparent in 2000 than 2007 because there were more of them in 2000.

We asked students if they had *ever* paid a bribe to pass an examination. We did not ask when they last paid such a bribe. The finding that a higher proportion of students in year four (or of those who had been in the medical school for five years or more) had paid to pass an examination does not necessarily mean that, in a given academic year, those students in their fourth year paid bribes for examinations more than students in lower years. It could simply be that students in their fourth year have had more opportunities to pay for examinations than those in their first to third year. It could also reflect a decreasing trend of paying for examinations over time. However, there is reason to believe that students have more incentive to pay for passing their fourth year examinations, which play an important part in determining their future. Hrabak reported that advanced students were more likely to use improper influence or to pay for passing an examination [9].

### Levels of corruption

Little has been reported about unofficial payments in medical schools and reported rates from the few published studies [8,9] might be only the tip of the iceberg. We focused on unofficial payments by students. These probably involve extortion on the part of the teacher, but the mechanisms of these corruption processes still need to be clarified.

In public universities the position of the teachers and administrative workers is similar to that of official government workers; in fact they *are* public workers. In this sense, the definition of corruption “the use of public position for personal gain” [26,27] applies to teachers and administrative workers in medical schools where corruption exists.

Considerable amounts of money may be involved in some of these corrupt transactions in the medical school. According to Kumar, in India, admission to medical school is so profitable that the transactions are patronised by politicians who take the enrolment payments as a source of income [20]. In Kenya the amount paid for bribes in public universities appears to be on the rise: those reported in 2003 were seven times higher than the previous year [28]. In Mexico, according to a newspaper report referred to earlier, each aspirant to a medical residency paid 80,000M$, the equivalent 1,580 days of work at the 2007 minimum wage, for the purchase of the admission test [11]. In our 2000 study, the average payment for admission into medical school was the equivalent to 149 days of work. We were unable to find comparable studies from other countries to compare the occurrence of corruption in the process of admission to medical schools.

### Perceptions about corruption

Our questionnaire did not ask about attitudes of the students toward unethical conduct although this was touched on in the focus groups. Other authors have found students’ attitudes to be a predictor of serious dishonest behaviour [9]. However, particularly in transactions where money is involved, the teacher shares responsibility. Probably the greater culpability is on the side of the teacher who, as a role model, is under greater obligation to avoid corrupt behaviour. Corruption is said to thrive where there is a monopoly of services, discretion in the application of the rules and low accountability [29]. All of these conditions could exist for medical school teachers. Particularly in small and medium-sized schools, like the one in our study, a specific subject is taught by only one, or a very few, teachers, creating an effective monopoly. The teachers determine the final grades and there is room for discretion. Teachers are accountable only to the head of the department or the school principal and that accountability is generally limited to their academic performance.

Our results showed an increased perception of corruption as an academic problem in the medical school in the second survey but without any increase in actual experience of corruption, and indeed a decrease in bribes to secure entry into the medical school. It is known that experiences of corruption tend to be under-reported because some respondents are reluctant to admit making unofficial payments [17,18]. Reluctance by students to report misconduct has been reported by other authors [8,9]. It is possible that students who completed the questionnaire in 2007 were more open to report (at least on the anonymous questionnaire) that they had paid a bribe than students in 2000, so there could in fact have been a reduction in the actual level of bribes for examination between 2000 and 2007, masked by this reporting difference. We do not know if this is so.

### Actions to tackle corruption

After the 2000 survey, the medical school implemented an admission test, conducted by an independent body, which all aspirants must pass in order to be admitted into the school. This is the main mechanism of control to avoid illegal enrolments. The proportion of students in the 2007 survey who reported paying for registration was indeed much lower than in the 2000 survey. The few students in the 2007 survey who paid for registration may have been deceived by corrupt staff members into believing they were enrolled by the influence of the staff member (who took their payment), when in fact they were admitted by the normal procedures. Only one first year student in the 2007 survey reported having paid for registration. The medical school plans to strengthen its information outreach about the official cost of the admission procedures and to emphasize that students should not make any other payments to obtain admission.

Collective examinations by department heads instead of by individual teachers were introduced in the medical school after the report of the 2000 survey was shared with the University Council. This was intended to decrease bribes to teachers for passing an examination. From our data, it is not apparent that this worked, although the low proportions of students in years one and two in 2007 reporting bribes to pass an examination are encouraging.

A study in the USA concluded that college students can play only a limited role in detecting teaching misconduct, and proposed strengthening of institutional codes of conduct and faculty peer sanctions [30]. Braxton and Bayer have argued for a formal code of ethics for undergraduate teaching [31]. Students’ focus groups in our study suggested disclosure, administrative penalties or dismissal for teachers involved in teaching malpractice. In another study, penalties for students for copying during examinations, including dismissal from the course, have been suggested [8]. However, given the disincentives for either party in corrupt transactions to report the event, it is likely that few cases will be detected, so the approach of the Autonomous University of Guerrero of changing the system of enrolment and examinations in order to avoid bribes, rather than to focus on punishment, seems appropriate.

## Conclusions

Some positives changes occurred in the medical school between the two surveys. The climate of political conflict improved, school facilities improved and student overcrowding was reduced. Student identification of their own low performance as a main academic problem in 2007 suggests that they became more self-critical. Still, student perceptions of corruption in the university remained high and weaknesses in the management of student services will require further improvement.

Medical schools need to build, in a deliberate way, an institutional culture of integrity. Even with scarce financial resources, the medical school in our study has made a commitment to reduce dishonest behaviour in the faculty. It appears that a more intensive and broader campaign is needed, involving university workers unions, the school’s academic council and students associations. Complete integrity in the medical school may take some years to achieve. Additional efforts are needed to reduce unethical behaviour of some teachers, specifically accepting bribes for passing an examination.

## Competing interests

The authors declare that they have no competing interests.

## Authors' contributions

AV, SP and JL designed the study. EN and JL conducted the field work. SP, JA and VD analysed and interpreted the data. SP, RL and AC wrote the manuscript. NA provided technical oversight and contributed to the final manuscript. All authors read and approved the final manuscript.
